# Changes in micro-relief during different water erosive stages of purple soil under simulated rainfall

**DOI:** 10.1038/s41598-018-21852-6

**Published:** 2018-02-22

**Authors:** Jian Luo, Zicheng Zheng, Tingxuan Li, Shuqin He

**Affiliations:** 1 College of Resources Science, Sichuan Agricultural University, 211 Huimin Road, Chengdu, Sichuan 611130 China; 2 College of Forestry, Sichuan Agricultural University, 211 Huimin Road, Chengdu, Sichuan 611130 China

## Abstract

This study investigated the variation characteristics of micro-topography during successive erosive stages of water erosion: splash erosion (SpE), sheet erosion (ShE), and rill erosion (RE). Micro-topography was quantified using surface elevation change, soil roughness (SR) and multifractal model. Results showed that the area of soil surface elevation decay increased gradually with the development of water erosion. With rainfall, the combined effects of the detachment by raindrop impact and the transport of runoff decreased SR, whereas rill erosion contributed to increase SR. With the increase in slope gradient, soil erosion area gradually decreased at the splash erosion stage. By contrast, soil erosion area initially decreased and then increased at the sheet and rill erosion stages. The width of the *D*_*q*_ spectra (Δ*D*) values increased at the splash erosion stage and then decreased at the sheet and rill erosion stages on the 10° slope, opposite to that on the 15° slope. The *ΔD* values decreased with the evolution of water erosive stages on the 20° slope. The slope had an enhancing effect on the evolution of water erosion. In this study, we clarified the essence of micro-topography and laid a theoretical foundation for further understanding diverse hydrological processes.

## Introduction

Micro-topography is defined by limited area with fewer changes in the relative elevation (usually not more than 5–25 cm). In general, there are four types of micro-topography. The first type is surface variations due to soil particles or soil aggregates and ranges in size from 0 to 2 mm. The second type is surface variations due to clods and ranges in size from 100 to 200 mm. The third type results from systematic differences in elevations due to tillage practices. The fourth type is surface elevation variations at the field level^1^. Micro-topography has been demonstrated a key parameter to influence penetration rate, depression storage, flow path connectivity, and soil erosion evolution^2–6^, and these effects are closely related to the spatial distribution of micro-topography. Due to the random nature and dynamic changes of micro-topography during rainfall, the effect of micro-topography on soil erosion is complicated and controversial, which indicates micro-topography may play a different role at the different water erosive stages.

In the past, many studies on micro-topography mainly focused on measuring methods, influencing factors, and the relationship between micro-topography and hydrological processes on slope^1,4^. However, there are few research focused on the spatial variability of micro-topography in the water erosion process, which is necessary for understanding diverse hydrological processes, as well as for modeling erosive processes. Indicators can be utilized to characterize the spatial heterogeneity of micro-topography. The surface elevation of micro-topography is controlled by a variety of factors including sediment transport, erosion and soil shrink-swell in the processes of water erosion^7^. Small-scale variations in surface elevation are of importance for the temporal and spatial variability of micro-topography. SR is a widely used index that represents the random distribution of micro-relief^8^. However, the effect of SR on soil erosion is somewhat uncertain. Some authors claim that the erosive power of runoff decrease as SR reduces flow velocity, which in turn reduced soil erosion^9,10^. However, some researchers found that SR can increase the soil erosive capacity by concentrating flows and the increased SR is a source of soil erosion^11^. In general, SR is defined as the height changes in reference to the typical shape of the soil surface micro-relief^12^. Fractal dimension can be used to analyze the associations between soil structure and other soil parameters^13^. The simple fractal dimension can provide approximate analysis of structured objects, while multifractal analysis (MFA) can effectively analyze spatial heterogeneity inside the structure. It has been widely applied in describing soil surface micro-relief on a micro-topography scale^8,14^, as well as geomorphology at basin scale^15^.

Purple soil is an important soil resource in China, covering 0.2 million km^2^ (about 2%) of its territory. They are characterized by lithologic soils without distinct pedogenic horizons and mainly distributed in Sichuan basin of Southwestern China and this soil is widely distributed throughout the world. Purple soil is a typical erodible soil due to its poor permeability of the underlying soil layer and heavy rainfall in Southern China^16^.

The object of this study was to investigate variation characteristics of micro-topography in the successive water erosive stages of splash erosion, sheet erosion and rill erosion under simulated rainfall, which would contribute to the understanding of the developmental process of slope erosion and reveal the essence of micro-topography of purple soil on sloping farmland.

## Results

### Variation characteristics of micro-topography

**Figure 1:**
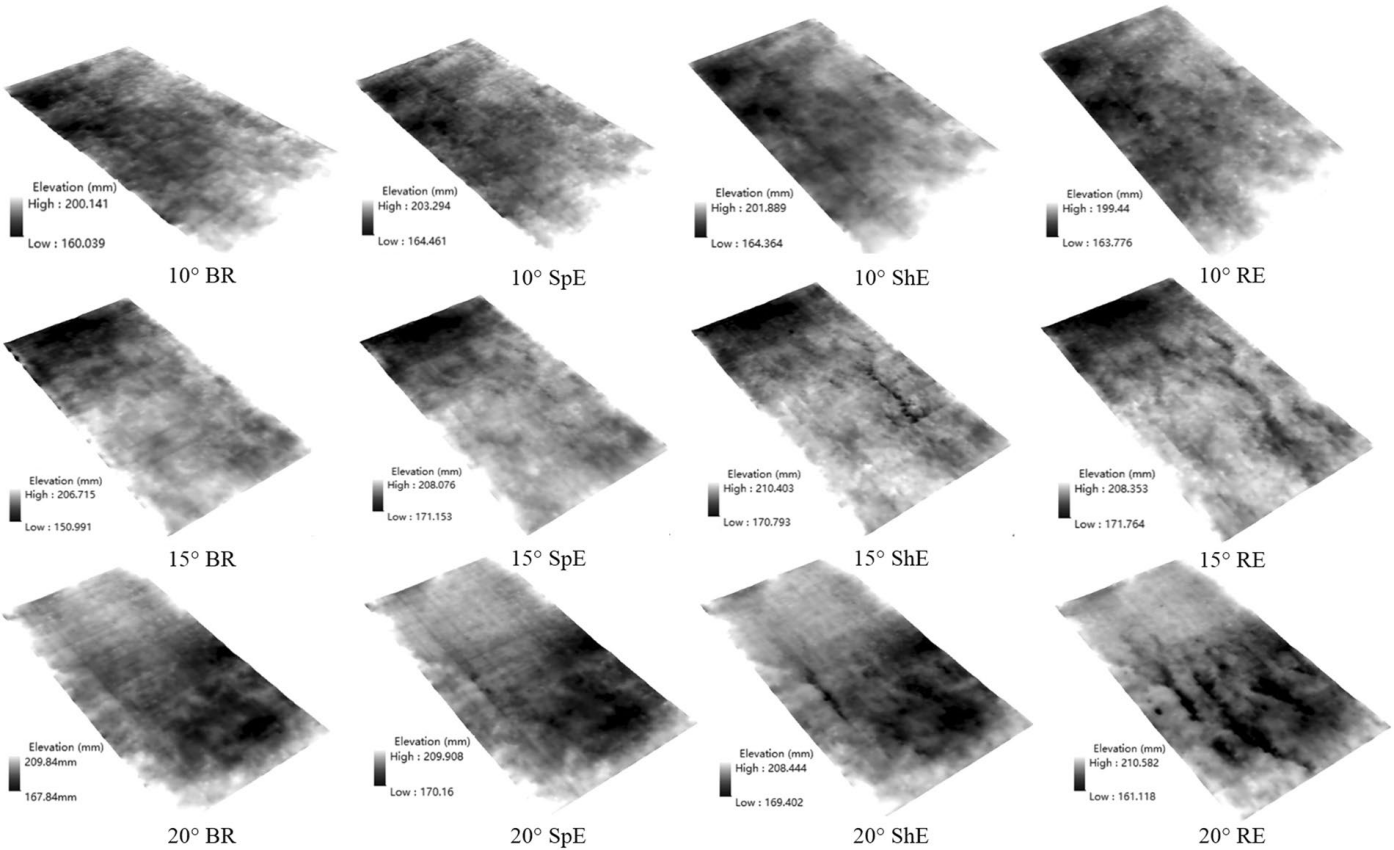
Change of micro-topography at different water erosive stages. Note: The BR indicates before rain, SpE, ShE and RE indicate splash erosion, sheet erosion and rill erosion, respectively. The same as below.

We modeled the micro-topographic changes at different erosive stages with increasing slope gradient (Fig. 1). Normally, the impact of raindrops detaches the soil, destroys soil structure, and decreases surface elevation. However, this study adopted air-dried soil with a low moisture content, the soil particles swelled with combined rainwater, thereby increasing the soil surface elevation at the splash erosion stage. Compared with the before rain stage, the elevation difference of slope gradients of 10°, 15°, and 20° decreased by 1.27, 1.89, and 2.25 mm, respectively. This finding indicated that the spurting effect of raindrops on soil aggregates was gradually strengthened from the 10° to the 20° slope. At the sheet erosion stage, it was relatively flat on the 10° slope. Soil surface loose materials transported with sheet flow, which formed many intermittent rills on the midslope and downslope of the 15° and 20° slopes. At the rill erosion stage, two rills generated on the midslope of the 10° slope (Fig. 1). The morphology of rills presented poor continuity and shallow depth, which led to the disappearance of rills at the end of rainfall. The rills developed obviously on 15° and 20° slopes.

In this study, the variation of soil surface elevation could be divided into <−6 mm, −6 mm to −4 mm, −4 mm to −2 mm, −2 mm to 0 mm, 0 mm to 2 mm, 2 mm to 4 mm, 4 mm to 6 mm, and >6 mm (i.e., grades I to VIII) on the basis of the digital elevation model (DEM) (Table 1). The relative elevation is less than 0 mm, indicating that soil erosion has occurred in the area. More the sum of proportion of grade I, II, III and IV, more area will be exposed to soil erosion on the slope. At the splash erosion stage, the variation of soil surface elevation mainly concentrated in grade VII, with percentage between 44.21% and 51.04%, followed by grade VI under different slope gradients. At the sheet erosion stage, the variation in surface elevation mainly concentrated in grade V, with percentage between 44.98% and 49.03%, followed by grade IV. At the rill erosion stage, the variation of soil surface elevation mainly concentrated in grade IV, with percentage between 41.93% and 58.10%, followed by grade V. On the 10° slope, the proportion of the area of soil surface elevation decay were 1.72%, 48.97%, and 78.09% for respectively splash erosion stage, sheet erosion stage, and rill erosion stage. On the 15° slope, the proportion of the area of soil surface elevation decay were 0.51%, 26.56%, and 53.72% for, respectively, splash erosion stage, sheet erosion stage, and rill erosion stage. While on the 20° slope, the proportion of the area of soil surface elevation decay were 0.41%, 45.76%, and 72.99% for respectively splash erosion stage, sheet erosion stage, and rill erosion stage. As such, the area of soil surface elevation decay increased gradually with the evolution of erosive stages. In addition, the area of soil surface elevation decay decreased with the increase of slope gradients at the splash erosion stage; an increasing first and then decreasing trend can be observed at the sheet erosion stage and rill erosion stage.

**Table 1:** Grade distribution of surface elevation variation at different erosive stages.

Slope	Erosive stage	Grade of elevation variation							
		I	II	III	IV	V	VI	VII	VIII
10°	SpE	0.08	0.09	0.22	1.33	7.33	29.73	44.21	17.02
	ShE	0.28	0.88	6.00	41.81	44.98	5.75	0.29	—
	RE	0.16	1.77	18.06	58.10	20.54	1.17	0.17	0.02
15°	SpE	—	0.03	0.10	0.38	4.53	32.51	50.15	12.30
	ShE	0.95	1.35	4.78	19.48	48.78	22.19	2.08	0.39
	RE	0.76	2.07	8.96	41.93	32.91	10.67	1.90	0.79
20°	SpE	0.01	0.01	0.04	0.35	3.66	32.78	51.04	12.11
	ShE	0.18	0.72	4.00	40.86	49.03	4.67	0.46	0.08
	RE	4.82	5.06	16.3	46.81	24.25	2.59	0.17	0.01

### Variation characteristics of SR

**Figure 2:**
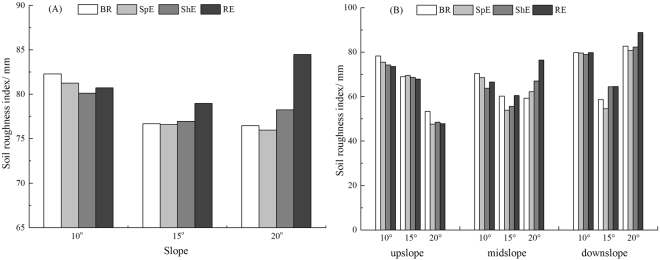
Change characteristics of soil roughness in entire slope (**A**) and different position (**B**) at different erosive stages.

As shown in Fig. 2, higher slope gradient presents more obvious difference in SR at different erosive stages. At the splash erosion stage, SR gradually decreased compared with the before rain stage under the condition of different slope gradients. At the sheet erosion stage, SR decreased on the 10° slope and increased on the 15° and 20° slopes compared to the splash erosion stage, and the change amplitude of SR were −1.39%, 0.46%, and 2.98% for respectively 10°, 15°, and 20° slope. At the rill erosion stage, SR increased compared to the sheet erosion stage, the change amplitude of SR were 0.74%, 2.62%, and 7.95% for respectively 10°, 15°, and 20° slope.

SR demonstrated a significant heterogeneity in different slope positions (Fig. 2). SR gradually decreased on the upslope with the evolution of water erosion, and such changes were complicated on the midslope and downslope. SR decreased at the splash erosion stage compared with that of the before rain stage in different slope positions. At the sheet erosion stage and rill erosion stage, SR decreased on the upslope. By contrast, SR increased on the midslope and downslope.

### Multifractal characteristics of micro-topography

**Figure 3:**
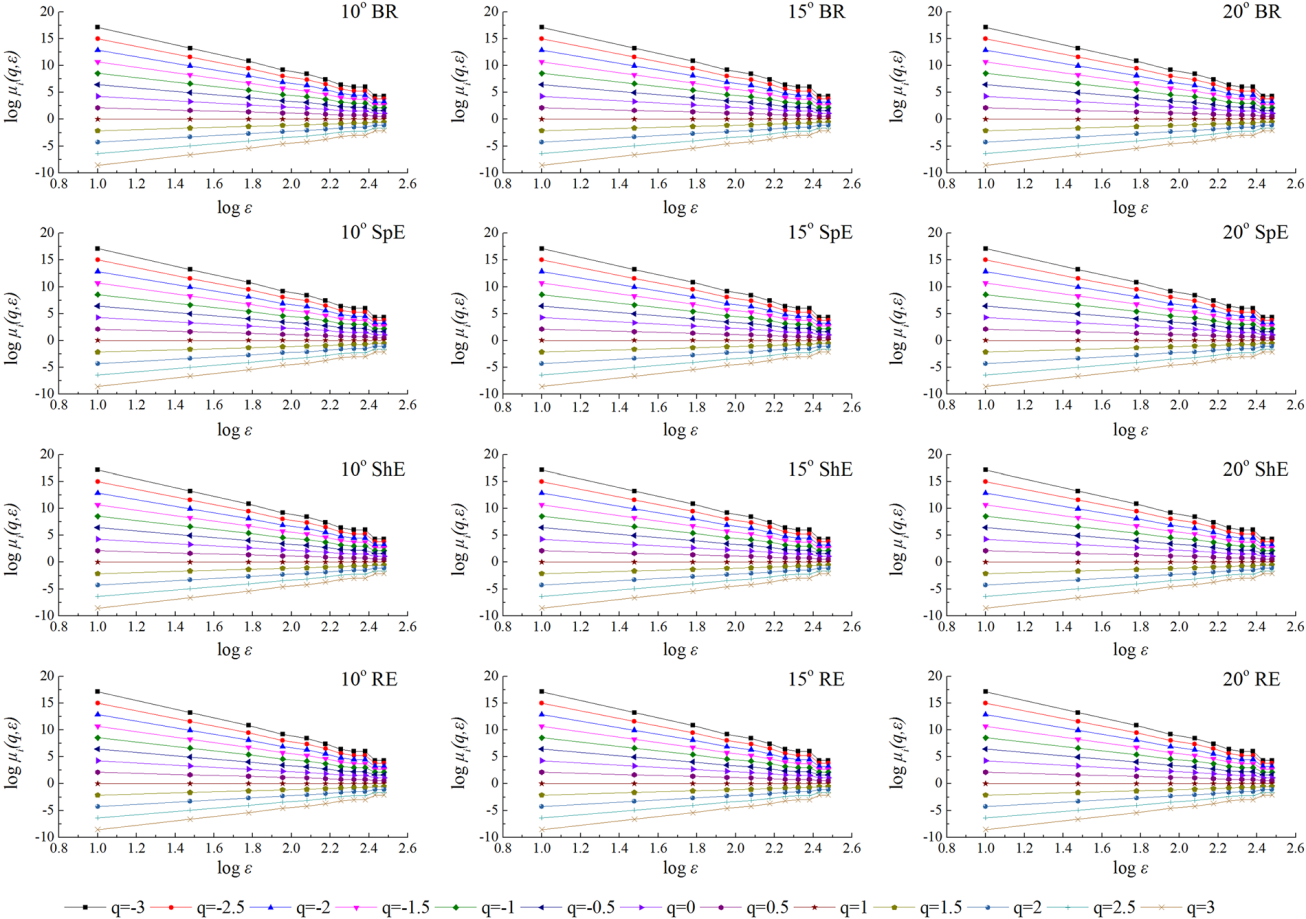
Relationship between log *µ*_*i*_(*q*, *ε*) and log *ε* of surface elevation at different erosive stages.

MFA is utilized to describe the singular distribution features of the quantity or status of a complex system. One of the most important steps in MFA is to determine the range of log *µ*_*i*_(*q*, *ε*) and log ε exhibiting linear behavior. Equations (6) and (7) were utilized to calculate the partition function. The double logarithmic graph of the partition function *µ*_*i*_(*q*, *ε*) and box dimension *ε* was thus obtained (Fig. 3). As shown in Fig. 3, log *µ*_*i*_(*q*, *ε*)-log ε fits the linear relation in the range of weighting factor *q* at different water erosive stages. The values of the determination coefficient were higher than 0.994. This finding indicated that soil micro-relief exhibited good scale invariance and possessed the characteristic structure of a multifractal under different water erosive stages.

**Figure 4:**
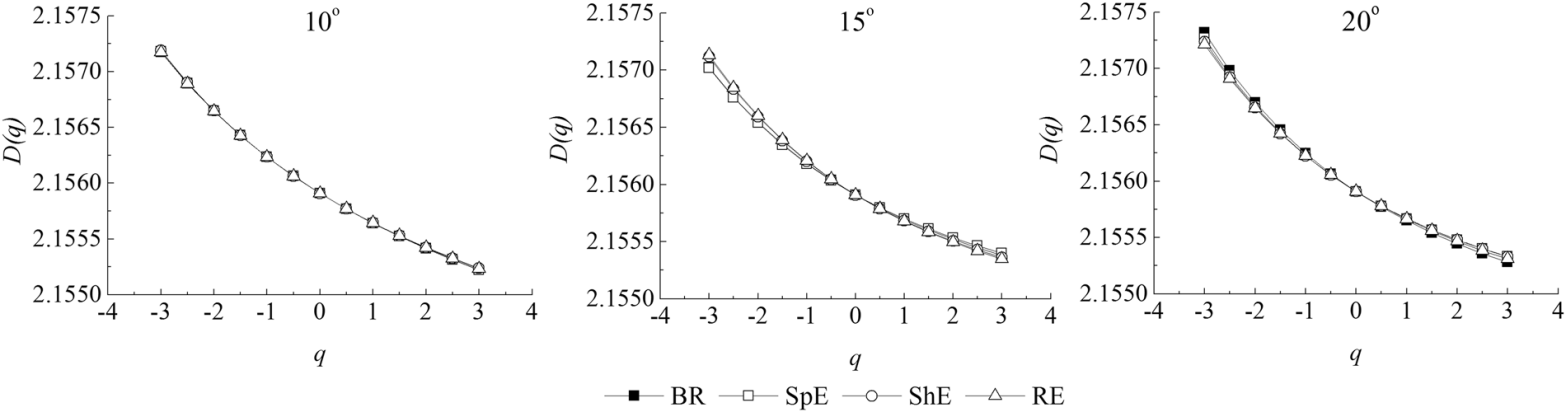
Relationship between *D*(*q*) and *q* of surface elevation at different erosive stages on different slope gradients.

As shown in Fig. 4, the general fractal dimension *D* values of micro-topography between 2.1550 and 2.1575 during successive erosive stages, and these *D* values monotonically decreased with the increase in *q*. In general *ΔD*, i.e. the width of the *D*_*q*_ spectra, indicated various degree in the heterogeneity of micro-topography. A larger *ΔD* is associated with a higher heterogeneity of micro-topography. Compared with the before rain stage, the change amplitude of *ΔD* values were 0.10%, −0.11%, and −0.76% for respectively splash erosion stage, sheet erosion stage, and rill erosion stage on the 10° slope. The change amplitude of *ΔD* values were −1.45%, 6.97%, and 8.55% for respectively splash erosion stage, sheet erosion stage, and rill erosion stage on the 15° slope. While the change amplitude of *ΔD* values were −5.17%, −6.41%, and −6.72% for respectively splash erosion stage, sheet erosion stage, and rill erosion stage on the 20° slope. Thus, on the 10° slope, the spatial variability of micro-topography increased at the splash erosion stage but gradually decreased at the sheet erosion stage and rill erosion stage. Conversely, the spatial variability of micro-topography of the 15° slope decreased at the splash erosion stage but gradually increased at the sheet erosion stage and the rill erosion stage. The spatial variability of micro-topography gradually decreased on the 20° slope with the evolution of water erosion.

**Figure 5:**
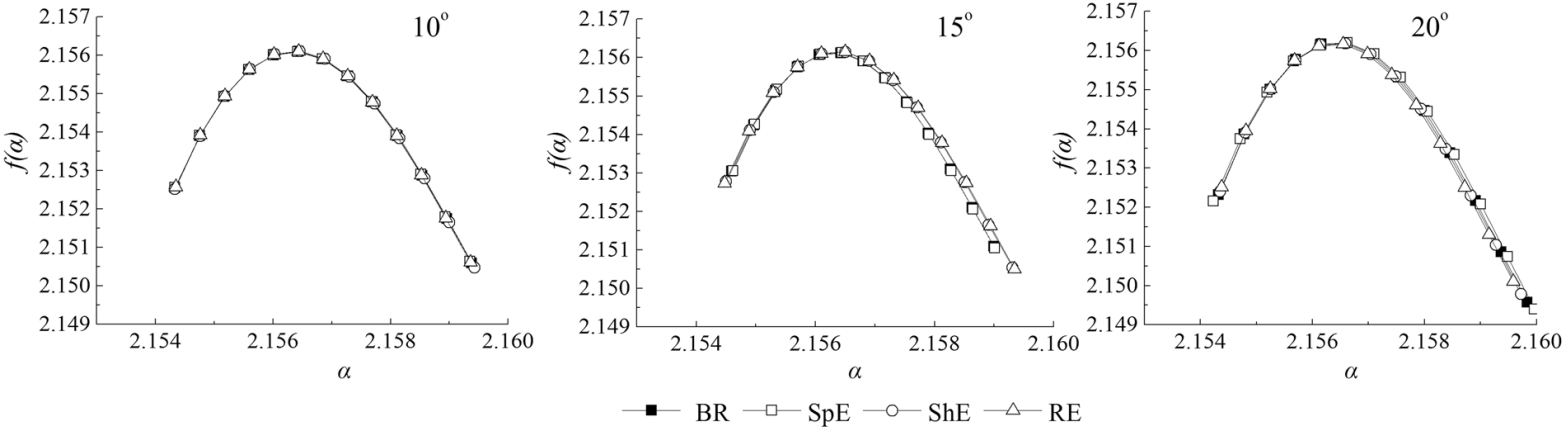
MFS of surface elevation at different erosive stages on different slope gradients.

We did the multifractal spectrum (MFS) of the different erosion stages on the different slopes (Fig. 5). The parameters of MFS are listed in Table 2. *f*(*α*)_max_ can be regarded as simple fractal dimension, which is a parameter that describing the overall characteristics of soil micro-relief. *f*(*α*)_max_ did not change with the evolution of erosive stages on the different slopes. This finding indices that the simple fractal suffered from certain limitations. *Δα* refers to the singular index span, which can quantitatively describe the heterogeneity degree of elevation distribution probability; a larger *Δα* indicates higher micro-relief (Moreno *et al*.^14^). In this study, *Δα* of the splash erosion stage increased by 1.51% compared with that of the before rain stage on the 10° slope, whereas no changes occurred at the other erosive stages. *Δα* of the splash erosion stage was similar to that of the before rain stage on the 15° slope. *Δα* increased by 9.52% rapidly at the sheet erosion stage but slightly increased at the rill erosion stage. We also observed that *Δα* gradually increased with the development of water erosion on the 20° slope. *Δf* (*α*) refers to the difference of MFS. *Δf* (*α*) ranged in the order of 20° > 15° > 10° at the different erosion stages. On the 10° slope, *Δf* (*α*) slightly increased at the splash erosion stage, whereas no changes occurred at the other erosive stages. On the 15° slope, *Δf* (*α*) gradually increased at the splash erosion stage and sheet erosion stage but remained steady at the rill erosion stage. On the 20° slope, *Δf* (*α*) gradually decreased with the development of water erosion.

**Table 2:** MFA of micro-relief characteristics at different erosive stages.

Slope	Erosive stage	*f*(*α*)_*max*_	*α*_*max*_	*α*_*min*_	*f*(*α*_*max*_)	*f*(*α*_*min*_)	*Δα*	*Δf*(*α*)
10°	BR	2.1561	2.1594	2.1543	2.1506	2.1526	0.0050	0.0019
	SpE	2.1561	2.1594	2.1543	2.1506	2.1525	0.0050	0.0020
	ShE	2.1561	2.1594	2.1543	2.1505	2.1525	0.0051	0.0020
	RE	2.1561	2.1594	2.1543	2.1506	2.1526	0.0050	0.0020
15°	BR	2.1561	2.1590	2.1546	2.1511	2.1530	0.0044	0.0019
	SpE	2.1561	2.1590	2.1546	2.1510	2.1531	0.0044	0.0020
	ShE	2.1561	2.1593	2.1545	2.1505	2.1528	0.0048	0.0022
	RE	2.1561	2.1593	2.1545	2.1505	2.1527	0.0049	0.0022
20°	BR	2.1562	2.1600	2.1542	2.1494	2.1521	0.0057	0.0028
	SpE	2.1562	2.1598	2.1543	2.1496	2.1523	0.0055	0.0027
	ShE	2.1562	2.1597	2.1544	2.1498	2.1524	0.0054	0.0026
	RE	2.1562	2.1596	2.1544	2.1501	2.1525	0.0052	0.0024

## Discussion

### Variation characteristics of micro-topography at the splash erosion stage

Splash erosion is a process of detachment and transportation of soil, and this phenomenon occurs mainly before runoff starts or at the beginning of runoff^17,18^. The effect of soil splash is co-determined by both soil and rainfall. Vertical impact stress distributions caused by raindrops are responsible for the shape of the raindrop impact crater^19^. The effect of splash erosion on different cohesive soils is relatively different. The manifestation of the raindrop impact stress may be ascribed to the soil collapse and sediment transport on non-cohesive soil surface, but it is mainly expressed in soil collapse on cohesive soil; moreover, the potholes caused by rainfall splash are relatively small and hard to develop. Purple soil is a soil type with high cohesion to bind the soil particles. On the one hand, rainfall splash would cause more potholes in the surrounding uplift. On the other hand, air-dried soil swelled in rainwater. Both effects led to the increase in soil surface elevation.

In this study, SR gradually decreased under the different slope gradients at the splash erosion stage compared with the before rain stage. This finding showed that the variation range of soil surface elevation decreased although the soil surface elevation increased. Interestingly, soil particles swelled in water; the splash of soil particles transferred and deposited in the potholes and thus increased the elevation values of surface depression. The superposition of two aspects decreased the variation range of soil surface elevation.

Soil surface elevation and SR reflected the variation of micro-topography in the vertical direction, whereas the multifractal parameters revealed the spatial variability of the micro-topography. As shown in Table 2, the *Δα* of the splash erosion stage was consistent with that of the before rain stage on the 10° and 15° slopes, but *Δα* decreased compared with the before rain stage on the 20° slope. This finding was attributed to the denser drainage networks on the lower slope gradient; the overland flow acts as a buffer against raindrop splash, and it can easily form the sparser drainage networks and deeper flow paths on the higher slope gradient, which is conducive to drainage^20^. Therefore, the effect of raindrop splash on micro-topography increased with the increase in slope gradient.

### Variation characteristics of micro-topography at the sheet erosion stage

Sheet erosion is a type of overland flow erosion, in which the soil is only eroded and transported from the surface of the profile^21^. Moss and Green showed that the overland flow can increase the sediment transport caused by raindrop impact^22^, and the effect of raindrop on the soil surface would be reduced when the water depth increases to the critical depth. Therefore, the sheet erosion took a leading position once the surface runoff formed. The response mechanism of the surface elevation was different between the splash erosion and the sheet erosion. At the splash erosion stage, the raindrop impact can act on the change in surface elevation directly in the vertical direction, with almost no soil loss on the entire slope (Table 1). At the sheet erosion stage, the soil surface was covered with a certain depth of water when the overland flow generated on the slope, and the raindrops acted on the overland flow directly and simultaneously affected the soil particles indirectly. Therefore, the soil particles transferred along the runoff path with the subsequent elevation change of fixed point on the soil surface.

In this study, SR of the upslope gradually decreased at the sheet erosion stage compared with the splash erosion stage under the different slope gradients, whereas SR of the midslope and downslope increased. This result can be attributed to the significant effect of slope position on SR during rainfall erosion^23^. When the runoff was formed, the overland flow on the upslope was mainly the sheet flow, which cannot separate soil efficiently, and the soil crust formed on the surface^24^. The overland flow on the midslope and downslope was mainly the stream, which caused the development of rills.

As presented in Table 2, the *Δα* of the sheet erosion stage increased compared with that of the splash erosion stage on the 10° and 15° slopes but decreased on the 20° slope mainly because the effect of runoff on the surface increased with the increase in slope gradient within a certain range. When the slope gradient exceeded the certain value, the interaction time between runoff and soil surface decreased. For one point on the surface, the runoff impulse decreased, which caused soil particles to not meet the starting condition of the sediment. The sediment was not transported by runoff, and the micro-relief was relatively low.

### Variation characteristics of micro-topography at the rill erosion stage

In general, rill erosion is the effect of flowing water exceeding a certain threshold of soil resistance^25^. The catchment area on the upslope increased with the increase in rainfall time and precipitation. The concentrated runoff caused shearing damage, thereby flowing the section soil and forming remarkable waterways and rill erosion. The development of rill is the result of a complex interaction of soil properties^26^. The purple soil used in this study was classified as a clay loam with about 22% clay particles and the distribution of clay minerals indicates the characteristics of endodynamorphic soil and soil-forming process^27^. The much more clay particles in the purple soil had much more capability to bond soil particles together to form micro or macro aggregates, which make higher micro-relief. Rill erosion development can be divided into two parts, namely, cutting down and widening^28^, which can increase SR. SR of the rill erosion stage was higher than that of the other erosion stages with the increase in slope gradient.

At the other erosion stages, the amplitude presented for both MFS was less pronounced, while a more robust spectrum at the rill erosion stage indicating more complexity in the soil structure. Zhang *et al*. stated the fractal dimension of micro-topography at various erosion stages of tilled loess slopes ranged from 1.54 to 1.86^6^. In this study, the fractal dimension of micro-topography at various erosion stages ranged from 2.1561 to 2.1562. This phenomenon illustrates that the spatial structure of sloping farmland of purple soil is more complex than that of loess at a smaller scale.

The symmetry of the MFS obtained on the two sites also highlights differences in the soil structure^14^. This difference is qualified *f*(*α*)values for positive and negative *q* with respect to its maximum. When Δ*f*(*α*) > 0, the number of maximum probability subsets is more than that of the minimum probability subsets, and soil micro-relief tends to be round. When Δ*f*(*α*) < 0, the number of minimum probability subsets is more than that of the maximum probability subsets, and soil micro-relief tends to be sharp^15^. As shown in Table 2, the spatial difference of micro-topography was not obvious on the 10° slope; the spatial variation of micro-topography increased, and micro-relief tended to be round on the 15° slope; finally, the spatial variation of micro-topography decreased, and micro-relief tended to be sharp on the 20° slope.

## Materials and Methods

### Study area and experimental soil

**Figure 6:**
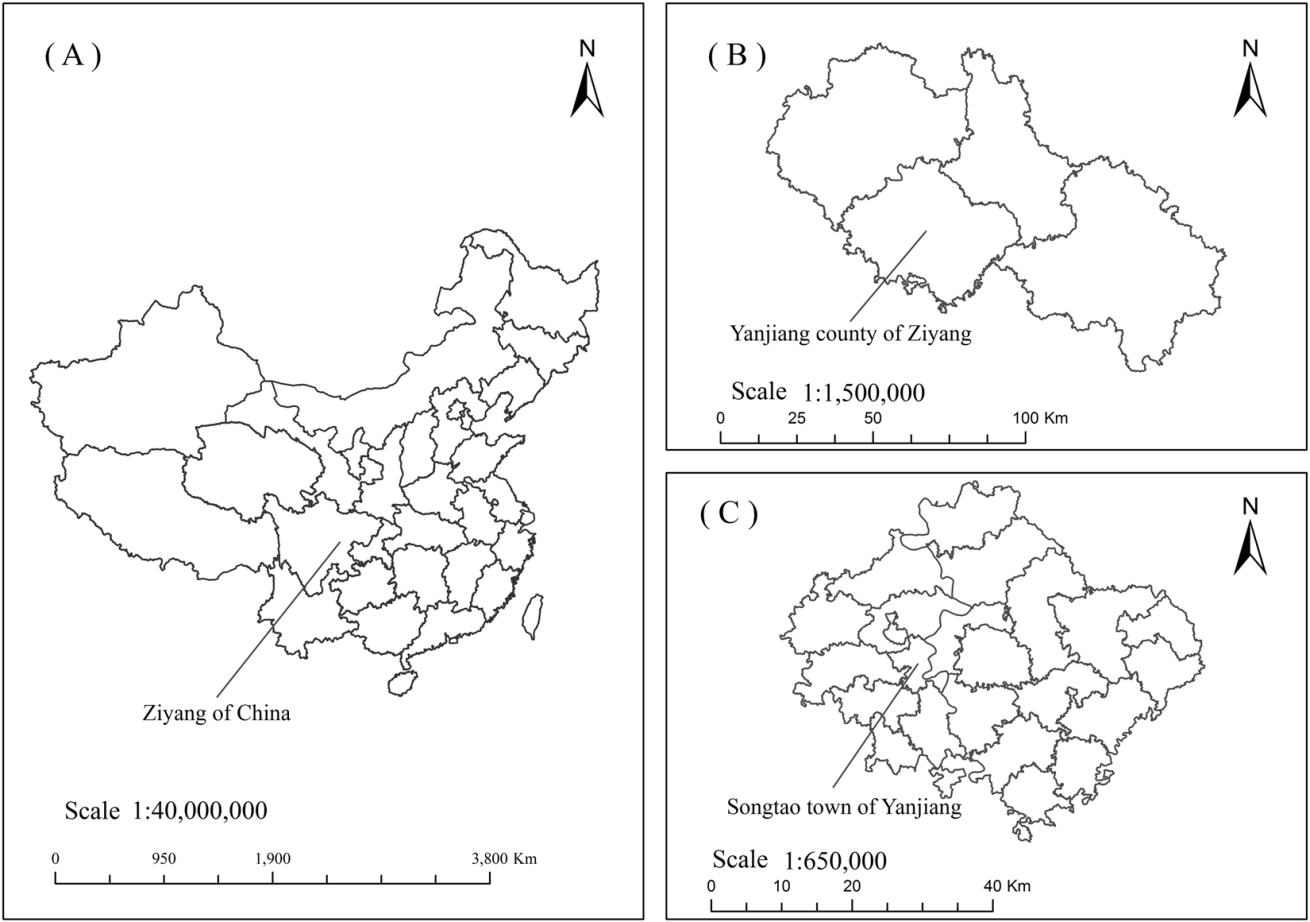
Location of the study area. Note: This map was made by ArcGIS 10.0 (http://www.esri.com/arcgis/about-arcgis).

Soil samples were taken from a field site in the upper reaches of the Huajiao River watershed, in the Yangtze River (104° 34′ E, 30° 05′ N, Fig. 6). The soil type in the area is purple soil (clay loam soil). The fractions of clay, silt, and sand were 22%, 29%, and 49%, respectively. The pH was 7.5, while the organic matter content was 7.3 g kg^−1^ (Table 3). This experimental area experiences a subtropical monsoon climate. The average annual rainfall is 980 mm, mainly concentrated in summer.

**Table 3:** Physical-chemical property of experimental soil.

Soil type	pH	Bulk density	CEC	SOC	CaCO_3_	Sand	Silt	Clay	Texture
		(g cm^−3^)	(cmol kg^−1^)	(g kg^−1^)	(%)	(%)	(%)	(%)	
Purple soil	7.5	1.2	21	13	12	49	29	22	Clay loam

Two iron boxes (2.0 m × 1.0 m × 0.5 m) were used in the rainfall simulation study. The soil was air dried and passed through a 10 mm sieve to ensure soil particle homogenization. The soil bulk density was controlled at 1.2 g cm^−3^ through randomization to ensure that it resemble natural soil state^29^. Based on the experimental slope length, the slope was equally classified into three categories: upslope, midslope and downslope.

### Rainfall simulation

The simulated rainfall experiments were conducted at the University Soil Erosion Research Laboratory, Sichuan Province. This rainfall simulator including two spray nozzles (V-80100) can be set to any selected rainfall intensity ranging from 0.5 to 3.0 mm min^−1^ by adjusting the nozzle size and water pressure. The height of the rainfall simulator measured 7 m, with the entire effective rainfall area measuring approximately 48 m^2^.

Linear slopes were prepared with slope gradients of 10°, 15°, and 20°. A rainfall intensity of 1.5 mm min^−1^ is representative erosive rainfall in the study area. Water erosion process can be divided into three stages: (1) splash erosion: at the beginning of runoff; (2) sheet erosion: the appearance of the drop pit on the slope; (3) rill erosion: the fish-scale shaped pits and overfall form the rills^6^. There are three erosive stages and three slope gradients, all experiments were repeated two times.

### Micro-topography measurement techniques

**Figure 7:**
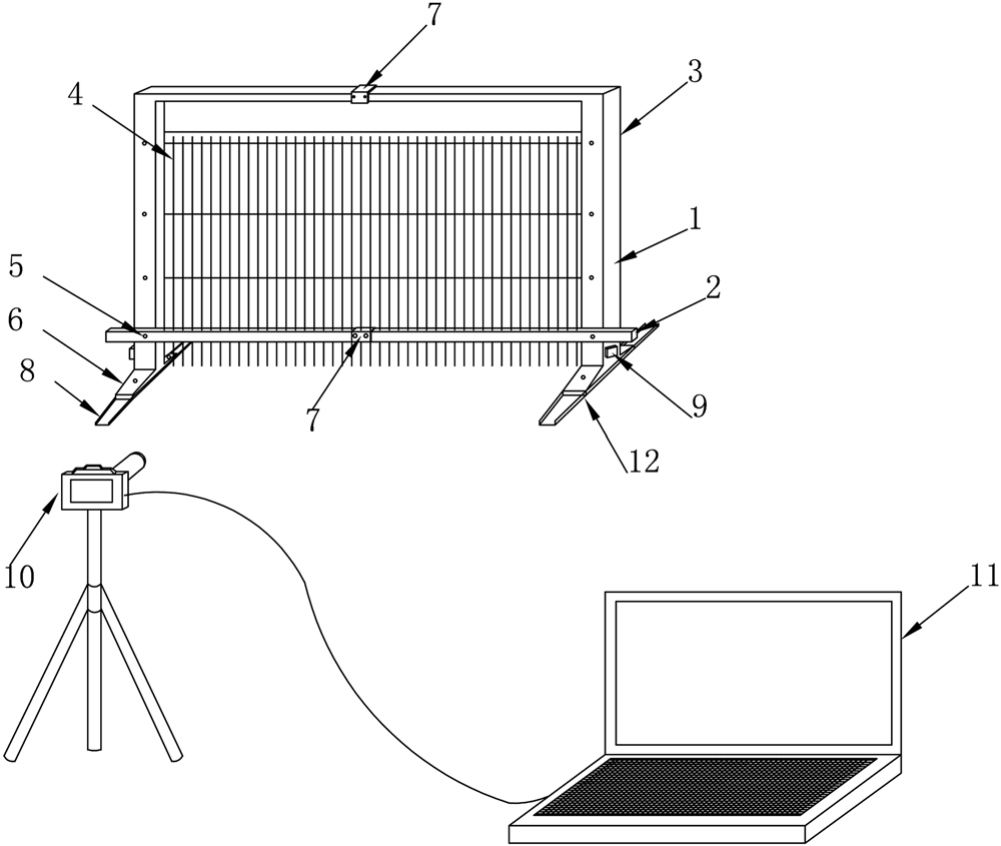
Scheme of the designed pin meter. Notes: 1. Support 2. Cross bar 3. Pin meter plate 4. Pin meter 5. Adjustment nut 6. Base 7. Widget 8. Level track 9. Micro motor 10. Camera 11. Output device 12. Extending parts. The figure was made by AutoCAD 2014 (https://www.autodesk.com/products/autocad).

The micro-topography measuring device used in this study were similar to those described by Luo *et al*.^30^. Soil surface micro-relief was measured using an improved pin meter (Fig. 7). The pin meter consisted of a single row of 50 pins, spaced at 2 cm intervals and measuring 50 cm in height. The pins were black, and a white background marked with 4 standard coordinate behind the pins, which helped automatically extract the coordinates of pins. Before measuring, the slope of soil box was adjusted to 0° and the pin meter plate was placed vertically on the soil box. A digital camera, Nikon D3000, was mounted on a tripod. The camera was placed directly in front of the soil box with the distance of 4.0 m. The pin meter moved forward 2 cm and a photo can be acquired. We can get 97 photographs in each soil box. Each photo was processed using homebrew to extract the pins vertex coordinates. A total of 4850 elevation data points were collected from the test area of each soil box at different rainfall stages, which provided the foundation for the construction of the micro-topographic digital elevation model and determined the changes of soil micro-relief during rainfall.

### Soil roughness analysis

Soil roughness index is one of the most widely used indicators for describing micro-topography^12^.

The SR index was calculated as follows Kuipers^31^:

1 $${\rm{SR}}=100\times \,\mathrm{log}\,{\rm{S}}$$

Where S is the standard deviation of all the surface elevations over an area of 2.0 m^2^.

### Multifractal analysis of micro-topography

#### Elevation distribution probability

In the calculation of multifractal spectra, the distribution probability of eigenvalues on the corresponding fractal structure should be first calculated. In this paper, the elevation value of each pixel is taken as the target, and the box covering method is used to calculate the elevation distribution probability, combined with the digital elevation model^15^. DEM is a commonly used model for describing terrain 3D information and it is a spatial data structure based on raster model. The box divides the whole space into square grids of equal size, called pixels. The elevation information is stored in the corresponding pixels in the form of attribute values.

A box with a dimension of ε × ε was used to cover the DEM of the entire tested area. To obtain the pixel elevation value in each box *H*_*i*_, which is divided by the sum of all pixel elevation values of all boxes in the entire area ∑*H*_*i*_, the elevation distribution probability in each box *µ*_*i*_ (ε) was obtained, the equations used for the calculation are as follows:

2 $${H}_{i}=\sum {h}_{mn}$$

3 $${\mu }_{{\rm{i}}}(\varepsilon )=\frac{{H}_{i}}{\sum {H}_{i}}$$

In this study, the pixel scale was set to 1 mm. The box dimension *ε* was set to 10, 30, 60, 90… 270, and 300 mm, respectively.

#### Scaling exponents

The set of all elevation distribution probability is expressed as a series of subsets, and satisfy the following relation:

4 $${\mu }_{{\rm{i}}}(\varepsilon )\propto {\varepsilon }^{{\alpha }_{{\rm{i}}}}$$

where *α*_*i*_ is the Holder index, which reflects the properties of each grid point set in which the elevation distribution probability *μ*_*i*_(*ε*) exhibits uniform scaling relations with *ε*. The larger the *α*_*i*_, the smaller the elevation distribution probability of the subset, and *α*_*i*_ ∈ [*α*_*min*_, *α*_*max*_].

*Na*_*i*_ (*ε*) is the number of boxes with a subset of the same *α*_*i*_ identity, it has a scaling relation in the scale-free region.

5 $$N{{\rm{a}}}_{{\rm{i}}}(\varepsilon )\propto {\varepsilon }^{-f({{\rm{a}}}_{{\rm{i}}})}$$

Where *f*(*α*_*i*_) is the fractal dimension of the grid point set of the same identity.

#### Moment analysis

The partition function *µ*_*i*_(*q*, *ε*) is the weighted summation of *q*th power of the distribution probability of elevation.

6 $${\mu }_{{\rm{i}}}(q,\varepsilon )={\sum _{i=1}^{{n}_{\varepsilon }}{\mu }_{{\rm{i}}}(\varepsilon )}^{q}$$

where *q* is the weight factor and *q* ∈ (−∞, +∞). The *q* values ranged from −3 to +3 with increments of 0.5, *n*_(*ε*)_ is the number of boxes in which *μ*_*i*_ (*ε*) > 0. For *q* → +∞, *µ*_*i*_(*q*, *ε*) reflects the properties of a subset with high probability, while for *q* → −∞, *µ*_*i*_(*q*, *ε*) reflects the properties of a subset with low probability.

In the scale-free region, *µ*_*i*_(*q*, *ε*) follows the scaling relation:

7 $${\mu }_{{\rm{i}}}({\rm{q}},\varepsilon )\propto {\varepsilon }^{\tau (q)}$$

where *τ*(*q*) is the scaling exponent value. The scaling exponent values were obtained by fitting the linear slopes of log–log plot of *µ*_*i*_(*q*, *ε*) versus ε.

8 $$\tau (q)=\mathop{\mathrm{lim}}\limits_{\varepsilon \to 0}\frac{\mathrm{ln}\,\sum _{i=1}^{n(\varepsilon )}{\mu }_{i}(q,\varepsilon )}{\mathrm{ln}(\varepsilon )}$$

The general fractal dimension *D*(*q*) can be obtained through a calculation based on the scaling exponent. The formula are as follows:

9 $$D(q)=\frac{1}{q-1}\mathop{\mathrm{lim}}\limits_{\varepsilon \to 0}\frac{\mathrm{ln}\,\sum _{i=1}^{n(\varepsilon )}{\mu }_{i}(q,\varepsilon )}{\mathrm{ln}(\varepsilon )},q\ne 1$$

10 $$D(1)=\mathop{\mathrm{lim}}\limits_{\varepsilon \to 0}\frac{\sum _{i=1}^{n(\varepsilon )}{\mu }_{i}(\varepsilon )\,\mathrm{ln}\,{\mu }_{i}(\varepsilon )}{\mathrm{ln}(\varepsilon )},q=1$$

Once a Legendre transformation for the *τ*(*q*)*-q* functions has been performed, the following equations can be utilized to calculate singularity index *α*(*q*) and dimension distribution function *f*(*a*)^32^.

11 $$\alpha (q)=\frac{d\tau (q)}{dq}$$

12 $$f(\alpha )=q\alpha (q)-\tau (q)$$

The *f*(*a*)~ *α*(*q*) function is the MFS.

## Conclusions

In general, micro-topography plays a different role at the different water erosive stages. At the splash erosion stage, the spatial heterogeneity of micro-topography was less multifractal behavior with lower SR index. At the rill erosion stage, the spatial heterogeneity of micro-topography presented stronger multifractal behavior with higher SR index. The spatial variation of micro-topography at the sheet erosion stage was between the splash erosion and the rill erosion. The slope had an enhancing role on the evolution of water erosion. The 20° slope had strong spatial heterogeneity and was more susceptible to erosion.

The methods used in this work satisfactorily represented the spatial heterogeneity of micro-topography. Surface elevation change and SR index reflected the overall situation of surface profile, while MFA further quantified the complexity and hierarchical spatial arrangement of micro-topography in a given situation. Therefore, both SR index and multifractal parameters should be taken into account when describing soil micro-relief.

It is expected that the results of this study could contribute to the quantitative classification of erosion stages according to the characteristic values of SR index and MFS. However, the experiment in this study considered a single rainfall intensity of 1.5 mm min^−1^ within a certain area of 2.0 m^2^. Further study is needed on the spatial and temporal variability of micro-topography under different rainfall intensities and tillage measures.

## References

[1] Zhang Q, Zhao L, Wang J, Wu F (2014). Spatiotemporal variability and simulation of tillaged loess microtopography in water erosion. J. Soil Water Conserv..

[2] Kirkby M (2002). Modelling the interactions between soil surface properties and water erosion. Catena.

[3] Morbidelli R (2015). Infiltration on sloping surfaces: laboratory experimental evidence and implications for infiltration modeling. J. Hydrol..

[4] Planchon O, Darboux F (2002). A fast, simple and versatile algorithm to fill the depressions of digital elevation models. Catena.

[5] Darboux F, Davy P, Gascuel-Odoux C, Huang C (2002). Evolution of soil surface roughness and flowpath connectivity in overland flow experiments. Catena.

[6] Zhang Q (2015). Spatial heterogeneity of surface roughness during different erosive stages of tilled loess slopes under a rainfall intensity of 1.5 mm min^−1^. Soil Till. Res..

[7] Wang GD, Wang M, Lu XG, Jiang M (2016). Surface elevation change and susceptibility of coastal wetlands to sea level rise in Liaohe Delta, China. Estuar. Coast. Shelf Sci..

[8] Martínez FSJ, Caniego J, Guber A, Pachepsky Y, Reyes M (2009). Multifractal modeling of soil microtopography with multiple transects data. Ecol. Complex..

[9] Cogo NP, Moldenhauer WC, Foster GR (1984). Soil loss reductions from conservation tillage practices. Soil Sci. Soc. Am. J..

[10] Zheng ZC, He SQ, Wu FQ (2014). Changes of soil surface roughness under water erosion process. Hydrol. Process..

[11] Römkens MJM, Helming K, Prasad SN (2002). Soil erosion under different rainfall intensities, surface roughness, and soil water regimes. Catena.

[12] Vermang J (2015). Characterization of soil surface roughness effects on runoff and soil erosion rates under simulated rainfall. Soil Sci. Soc. Am. J..

[13] Bird N, Díaz MC, Saa A, Tarquis AM (2006). Fractal and multifractal analysis of pore-scale images of soil. J. Hydrol..

[14] Moreno RG, Álvarez MCD, Alonso AT, Barrington S, Requejo AS (2008). Tillage and soil type effects on soil surface roughness at semiarid climatic conditions. Soil Till. Res..

[15] Shen ZY, Li ZB, Li P, Lu KX (2009). Multifractal arithmethic for watershed topographic feature. Advances in Water Science.

[16] Chen XY, Huang YH, Zhao Y, Mo B, Mi HX (2015). Comparison of loess and purple rill erosions measured with volume replacement method. J. Hydrol..

[17] Ma B (2015). Soil splash detachment and its spatial distribution under corn and soybean cover. Catena.

[18] Mahmoodabadi M, Sajjadi SA (2016). Effects of rain intensity, slope gradient and particle size distribution on the relative contributions of splash and wash loads to rain-induced erosion. Geomorphology.

[19] Nearing MA, Bradford JM, Holtz RD (1987). Measurement of waterdrop impact pressures on soil surfaces. Soil Sci. Soc. Am. J..

[20] Xie S, Cheng Q, Xing X, Bao Z, Chen Z (2010). Geochemical multifractal distribution patterns in sediments from ordered streams. Geoderma.

[21] Heung B, Bakker L, Schmidt MG, Dragićević S (2013). Modelling the dynamics of soil redistribution induced by sheet erosion using the universal soil loss equation and cellular automata. Geoderma.

[22] Moss AJ, Green P (1983). Movement of solids in air and water by raindrop impact. Effects of drop-size and water-depth variations. Soil Res..

[23] Zhao LS, Zhang QF, Wang J, Wu FQ (2013). Effect of soil surface roughness on rainfall erosion as affected by slope position on loess slope. Acta Pedologica Sinica.

[24] Bu CF, Wu SF, Yang KB (2014). Effects of physical soil crusts on infiltration and splash erosion in three typical Chinese soils. Int. J. Sediment Res..

[25] Wirtz S, Seeger M, Ries JB (2012). Field experiments for understanding and quantification of rill erosion process. Catena.

[26] Zhang P, Tang H, Yao W, Zhang N, LV XZ (2016). Experimental investigation of morphological characteristics of rill evolution on loess slope. Catena.

[27] Xiao Y, Tang J, Wang M (2016). Physicochemical properties of three typical purple soils with different parent materials and land uses in Sichuan Basin, China. Natural Resources and Engineering.

[28] Shen H, Zheng F, Wen L, Han Y, Hu W (2016). Impacts of rainfall intensity and slope gradient on rill erosion processes at loessial hillslope. Soil Till. Res..

[29] An J, Zheng F, Lu J, Li G (2012). Investigating the role of raindrop impact on hydrodynamic mechanism of soil erosion under simulated rainfall conditions. Soil Sci..

[30] Luo J, Zheng Z, Li T, He S (2017). Spatial heterogeneity of microtopography and its influence on the flow convergence of slopes under different rainfall patterns. J. Hydrol..

[31] Kuipers H (1957). A relief meter for soil cultivation studies. Neth. J. Agric. Sci..

[32] Sohnius MF, Stelle KS, West PC (1980). Dimensional reduction by Legendre transformation generates off-shell supersymmetric Yang-Mills theories. Nucl. Phys. B.

